# An Account of a Fatal Vomiting, Apparently Brought on by a Disease of the Kidney's

**Published:** 1786

**Authors:** William Keir

**Affiliations:** Physician to St. Thomas's Hospital


XVII. *An Account of a fatal Vomiting, apparently brought on by a Difeafe of the Kidneys. By the late* William Keir, *M. D. Phyfician to St. Thomas's Hofpital.* Vide *Medical Communications.* Vol. I. 8vo. Johnfon, *London*, 1785.

THE circumftance in this cafe, which appears chiefly to merit attention is, the vomiting connected with a difeafe of the kidneys, in which neither pain nor inflammation were concerned; and from which, as no other adequate caufe could be found, the vomiting is fuppofed to have arifen.

The patient was a woman thirty years old, and when our author firft faw her, more than a week had elapfed without any evacution by ftool, and during the laft two days fhe had been troubled with a vomiting of whatever fhe fwallowed. On examining her belly, a tumor, larger than a fift, was felt deeply feated in the right fide, nearly at an equal diftance between the cartilages of the laft falfe rib and the fpine of the ilium. This tumor, which, from the thinthinneſs of the patient, could be diſtinctly felt both before and behind, was perfectly circumſcribed and moveable in every direction. She had obſerved it ſix weeks, and it was ſo free from pain, that ſhe ſuffered no uneaſineſs when a perſon, who ſuppoſed it to conſiſt of hardened fæces, preſſed it ſo ſtrongly as to perſuade himſelf of its doughy conſiſtence, and of having left the impreſſion of his finger on its ſurface. Her ſkin was hot; her pulſe beat about one hundred and twenty ſtrokes in a minute, and her urine about this time was obſerved to be in ſmall quantities, and even when recently voided, to have a whitiſh turbid appearance. She could give no diſtinct account of any preceding diſorder, or of any cauſe to which her complaints might be aſcribed, except that her health had been bad for ſome time, and that ſhe had recovered but a few weeks before from an ague.

The treatment adopted in this caſe was founded on a ſuppoſition, that the ſymptoms originated from an accumulation of hardened excrement in the colon; but although ſtools were from time to time procured by means of clyſters, the vomiting, the tumor, and the other ſymptoms continued as before. About four or five days before her death, which which happened within eight days from the time our author firft faw her, fhe mentioned a flight uneafinefs towards the left fide of her navel, and a fmall degree of pain at the pit of the ftomach. From thefe, however, we are told, fhe feemed to fuffer but very little, for fhe fpoke of them only when fhe was ftrictly queftioned whether fhe felt any uneafinefs. The tumor already mentioned never feemed to be attended with pain.

During the laft two or three days of her life, her pulfe beat irregularly about forty ftrokes in a minute; fhe had a frequent hiccough; and was inclined to fleep much, but was eafily roufed, and free from delirium. Under thefe complaints fhe gradually funk and died.

On diffection, the tumor in the right fide of the abdomen was found to be the kidney of that fide greatly enlarged. Its furface, however, was fmooth and equal; and in cutting into its fubftance it feemed to be found. A few fmall, irregular calculi adhered to the membrane lining the pelvis of the kidney, the cavity of which contained a fmall quantity of bloody fluid. Blood alfo was found in the right ureter, which, in other refpects, was in its natural ftate. The right emulgent vein was fo so large as, when flattened, to meaſure more than three quarters of an inch in breadth. The correſponding artery was of the uſual ſize. Of the left kidney, which had a lobulated form, ſcarcely any thing ſeemed to remain but the inveſting membrane; which was filled with a ſubſtance like chalk reduced with water into the conſiſtence of thick paſte. Diſperſed through this were found ſome fragments of calculous matter, of the ſize of grapes ſtones. The urinary bladder was empty; and its ſubſtance was thicker than uſual; but in none of the urinary organs was the ſmalleſt trace to be perceived of inflammation. The reſt of the abdominal viſcera exhibited no mark of diſeaſe, if we except a few ſmall tubercles which were obſerved in the pancreas. In the thorax nothing preternatural was diſcovered.

The facts here related, afford, as the author judiciouſly obſerves, a proof of a cloſer and more extenſive ſympathy between the kidneys and ſtomach, than has generally been thought to ſubſiſt. It has long been known that the ſtomach may be much diſordered by diſeaſes of the kidneys, attended with inflammation, or with violent pain; but that a ſtate of thoſe organs accompanied with neither ſhould produce duce a fimilar effect, has not, he thinks, been commonly imagined.

This cafe likewife, the author remarks, may affift us to diftinguifh between difeafes of the inteftinal canal, and thofe of the kidneys; becaufe if ficknefs and vomiting fhould occur without pain, or any fign of inflammation, the caufe of the difeafe, even if conftipation fhould attend, might now, he thinks, with more reafon be fought for in the kidneys than in the inteftines. Thefe inferences, he candidly obferves, would be lefs allowable, if the prefent were the only inftance of its kind; but although certainly rare, it is, he adds, perhaps not entirely unexampled, as a cafe with fimilar fymptoms, and like this remarkable for the abfence of pain, is recorded by Morgagni *, who, from feveral circumftances (although the body was not examined by diffection) ventured to conclude that the caufe of the vomiting, in that inftance, was a difeafe of the right kidney.

---

XVIII. *Refult of fome Obfervations made by* Benjamin Rufh, *M. D. Profeffor of Chemiftry*

* De Sed. et Cauf. Morb. Lib. 3. Epift. 30. Art. 22.

